# Comparison between xCELLigence biosensor technology and conventional cell culture system for real-time monitoring human tenocytes proliferation and drugs cytotoxicity screening

**DOI:** 10.1186/s13018-017-0652-6

**Published:** 2017-10-16

**Authors:** Chih-Hao Chiu, Kin Fong Lei, Wen-Ling Yeh, Poyu Chen, Yi-Sheng Chan, Kuo-Yao Hsu, Alvin Chao-Yu Chen

**Affiliations:** 1 Department of Orthopedic Surgery, Chang Gung Memorial Hospital, Taoyuan, Taiwan; 2 Bone and Joint Research Center, Chang Gung Memorial Hospital, Linkou, Taiwan; 3 Ph.D. Program in Biomedical Engineering, College of Engineering, Chang Gung University, Taoyuan, Taiwan; 4 Graduate Institute of Medical Mechatronics, Chang Gung University, Taoyuan, Taiwan; 5 Department of Mechanical Engineering, Chang Gung University, Taoyuan, Taiwan; 6 Department of Radiation Oncology, Chang Gung Memorial Hospital, Linkou, Taiwan; 7 Department of Orthopedic Surgery, Chang Gung Memorial Hospital, Linkou, Taiwan; 8 Department of Occupational Therapy and Graduate Institute of Behavioral Science, Chang Gung University, Taoyuan, Taiwan

**Keywords:** Tendinopathy, Tenocytes, Cytotoxicity, XCELLigence, Microfluidic, Real-time screening, Ketorolac tromethamine, Bupivacaine, Methylprednisolone, Betamethasone

## Abstract

**Background:**

Local injections of anesthetics, NSAIDs, and corticosteroids for tendinopathies are empirically used. They are believed to have some cytotoxicity toward tenocytes. The maximal efficacy dosages of local injections should be determined. A commercial 2D microfluidic xCELLigence system had been developed to detect real-time cellular proliferation and their responses to different stimuli and had been used in several biomedical applications. The purpose of this study is to determine if human tenocytes can successfully proliferate inside xCELLigence system and the result has high correlation with conventional cell culture methods in the same condition.

**Methods:**

First passage of human tenocytes was seeded in xCELLigence and conventional 24-well plates. Ketorolac tromethamine, bupivacaine, methylprednisolone, and betamethasone with different concentrations (100, 50, and 10% diluted of clinical usage) were exposed in both systems. Gene expression of type I collagen, type III collagen, tenascin-C, decorin, and scleraxis were compared between two systems.

**Results:**

Human tenocytes could proliferate both in xCELLigence and conventional cell culture systems. Cytotoxicity of each drug revealed dose-dependency when exposed to tenocytes in both systems. Significance was found between groups. All the four drugs had comparable cytotoxicity in their 100% concentration. When 50% concentration was used, betamethasone had a relatively decreased cytotoxicity among them in xCELLigence but not in conventional culture. When 10% concentration was used, betamethasone had the least cytotoxicity. Strong and positive correlation was found between cell index of xCELLigence and result of WST-1 assay (Pearson’s correlation [*r*] = 0.914). Positive correlation of gene expression between tenocytes in xCELLigence and conventional culture was also observed. Type I collagen: [*r*] = 0.823; type III collagen: [*r*] = 0.899; tenascin-C: [*r*] = 0.917; decorin: [*r*] = 0.874; and scleraxis: [*r*] = 0.965.

**Conclusions:**

Human tenocytes could proliferate inside xCELLigence system. These responses varied when tenocytes were exposed to different concentrations of ketorolac tromethamine, bupivacaine, methylprednisolone, and betamethasone. The result of cell proliferation and gene expression of tenocytes in both xCELLigence and conventional culture system is strongly correlated.

**Clinical relevance:**

xCELLigence culture system may replace conventional cell culture, which made real-time tenocyte proliferation monitoring possible.

## Background

Tendinopathies are empirically treated with injections of anesthetics, non-steroids anti-inflammatory drugs (NSAIDs), and corticosteroids [1]. Bupivacaine, ketorolac tromethamine, methylprednisolone, and betamethasone are commonly used for myofascial pain [2, 3]. There are currently no available data regarding the maximum effective dosages of local peritendinous injections [3], especially regarding differences in patient gender, age, and stage of tendon injury. A good screening tool to determine the optimal dose of local injection is necessary.

Cell number is commonly quantified to explore cellular behavior under specific culture conditions. This is conventionally performed by directly counting cells under a microscope, detecting the turbidity of a cell suspension optically, or indirectly quantifying cellular components. These standard approaches require large numbers of cells, large volumes of reagents, and are limited in their accessibility for high resolution and time-lapse imaging [4]. Cell measurement based on detecting cellular components destroys the cells and thus prohibits subsequent cellular assays. These conventional endpoint assays facilitate cellular assessment only at defined times. Real-time monitoring is not possible, and assessing time-dependent effects is laborious and prone to mechanistic errors [5].

Hung et al. first developed a system with the ability to maintain and monitor cells continuously while providing a stable microenvironment called a microfluidic cell culture system [6]. With microfluidic technology, cells can be cultured and manipulated in a closed-volume environment to study cellular behavior [7]. This technology has been employed to create higher throughput analysis platforms of cell behavior compared to conventional techniques [8].

Biological substances can be quantified by detecting their electrical impedance. Electronic circuits are integrated into microfluidic chips to electrically record cellular responses [9, 10]. The working principle is based on the use of microelectrodes fabricated on a substrate surface that serves as an electrical transducer. Microelectrode surfaces are functionalized with biomolecules capable of recognizing the target analytes. When the target analyte is present on the microelectrodes, the analyte binds to the immobilized biomolecules, causing measureable changes in electrical impedance across the microelectrode. According to the quantitative relationship between impedance change and analyte concentration, the target analyte can be quantified. Cell populations that differ in their cell density, proliferation rate, adhesion characteristics, or cell morphology can be distinguished by the impedance readout [11].

Combining microfluidic cell culture techniques and real-time impedance measurement, a commercial 2-dimensional microfluidic cell analyzer called the xCELLigence system (xCELLigence, Roche/ACEA Biosciences, CA) was developed to monitor cellular responses. Real-time detection of cell death in immortalized hippocampal neurons (HT-22 cells), neuronal progenitor cells (NPC), and differentiated primary cortical neurons was successfully demonstrated using this system [12]. In orthopedic field, Scrace et al. [13] utilized this system to monitor rat tenocyte adhesion. This biosensor technology allows continuous real-time monitoring of cellular adhesion properties in vitro in a non-invasive, label-free manner [14].

We proposed that human tenocyte proliferation and their real-time responses to stimuli could be recorded by utilizing xCELLigence system. A good correlation could be found between xCELLigence readout and results of conventional cell proliferation assays. End-point results of cell proliferation and gene expression could be alike in both systems. We hypothesize that xCELLigence biosensor technology can serve as a valuable platform for real-time monitoring of tenocyte behavior and their responses to different stimuli, which can be adopted into clinical practice to help determine optimal doses of drugs when local injection is considered.

## Methods

Level of evidence: this is a level III controlled laboratory study.

### Isolation of human tenocytes

**Fig. 1:**
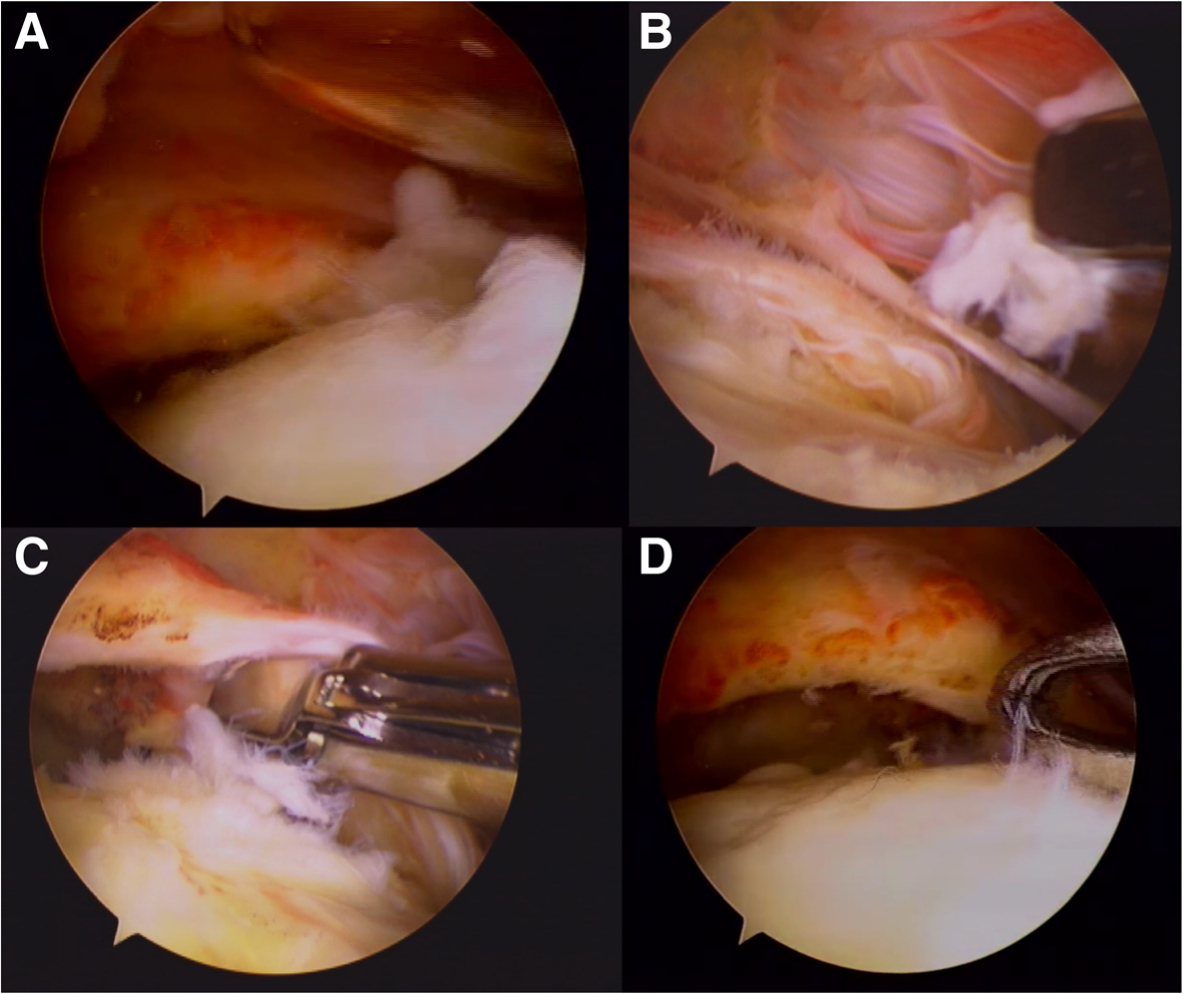
Images taken during harvest of the human supraspinatus tendon. **a** Identification of the torn supraspinatus. **b**, **c** Supraspinatus harvest. **d** After supraspinatus harvest

**Fig. 2:**
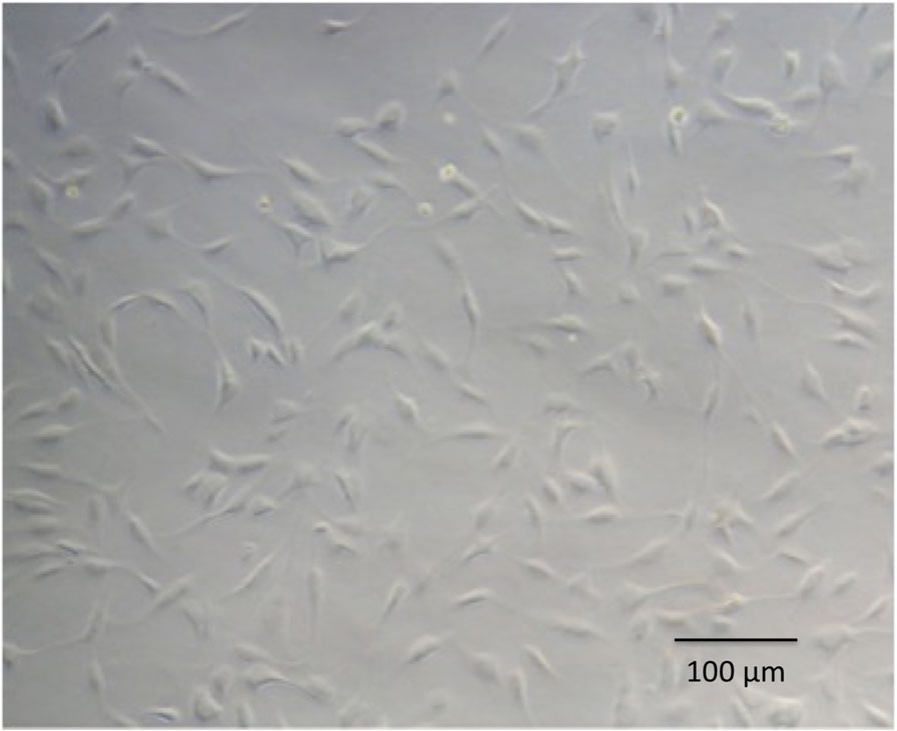
Spindle-like cells were observed in first-passage human tenocytes before cell seeding using a light microscopy

Human tenocytes were isolated from the torn edge of supraspinatus tendon of a 64-year-old female patient during arthroscopic repair (Fig. 1), which was approved by the Institutional Review Board at our Hospital of the first author (Chairman, Tsang-Tang Hsieh, M.D., Institutional Review Board, Chang Gung Medical Foundation). The written consent was informed before the surgery with patient herself. Tendon samples were digested in an enzymatic solution containing 4 mg/mL dispase (Roche, Burgess Hill, UK) and 300 U/mL collagenase type II (Gibco, Invitrogen, Paisley, UK) at 37.8 °C for 16 h. After digestion, the mixture was filtered and centrifuged at 1000 rpm (400×*g*) for 5 min at 37 °C. The cell pellet was then suspended and maintained in culture media (minimum essential medium; α-MEM) supplemented with 10% FBS and 1% antibiotics in standard tissue culture flasks. After the first passage, the adherent monolayer was trypsinized and cells were seeded at 2 × 10^4^ cells/cm^2^ in conventional 6-well plates and maintained in serum-free α-MEM overnight at 37.8 °C prior to loading in the microfluidic system or conventional 24-well plates. Normal tenocyte morphological characteristics were confirmed by microscopy (Fig. 2).

### Introduction of the xCELLigence system

The xCELLigence system (Roche/ACEA Biosciences, San Diego, CA) is a commercial microfluidic system designed to allow for continuous real-time monitoring of cellular adhesion properties in vitro in a non-invasive, label-free manner. The study was performed according to manufacturer’s instructions.

### Seeding tenocytes into xCELLigence E-96 plates

Firstly, we added complete media (50 μL) to wells of E-96 xCELLigence plates. After equilibration to 37 °C, plates were inserted into the xCELLigence station to measure the baseline impedance. This ensured that all wells and connections were working within acceptable limits. Four different tenocyte concentrations (5 × 10^3^ cells/cm^2^, 1 × 10^4^ cells/cm^2^, 2 × 10^4^ cells/cm^2^, and 4 × 10^4^ cells/cm^2^) were firstly seeded into the wells as a pilot study to determine the optimal amount of cell seeding for these experiments.

### Seeding tenocytes into conventional 24-well plates

In conventional 24-well plates, 2 × 10^4^ tenocytes/cm^2^ were seeded and were exposed to the same drug concentrations tested inside the xCELLigence cell culture system as a control group.

### Drug preparation and exposure

Three different concentrations of NSAIDs, anesthetics, and corticosteroids were added to the tenocytes in triplicate 24 h after seeding. For ketorolac tromethamine (Yung Shin Pharmaceutical) treatment, solutions of 30, 15, and 3 mg/mL (100, 50, and 10% of clinical dosage) were added to wells in a volume of 10 μL. For bupivacaine (Myungmoon Pharmaceutical), treatment, concentrations of 0.5, 0.25, and 0.05% were applied in a volume of 10 μL. For methylprednisolone (Pfizer Inc.) treatment, solutions of 40, 20, and 4 mg/mL were applied, and for betamethasone (Sinphar Pharmaceutical) treatment, solutions of 7, 3.5, and 0.7 mg/mL were used, all in a volume of 10 μL. Control cultures were exposed to a saline solution under the same conditions without anesthetics, NSAIDs, or steroids.

### xCELLigence software and data plotting

We used xCELLigence software version 1.2.1 in this experiment to provide an electronic record of the experimental details. The cell index represents the measure of cellular adhesion across each individual well. In the absence of living cells (media only) or with a suspension of dead cells, the cell index values will be close to zero. After cellular attachment onto the electrode, the measured signal correlates linearly with cell number throughout the experiment with sufficient accuracy, which has been shown in many publications [12, 14–18].

### Conventional cell proliferation assay: WST-1

Tenocytes (2 × 10^4^ cells/cm^2^) were seeded inside conventional 24-well plates and allowed to adhere overnight. The next day, cells were exposed to same drug treatments tested inside the xCELLigence system. The medium was changed every third day. Cell growth was analyzed on day 7 after drug exposure with a WST-1 kit (Roche, Basel, Switzerland). The WST-1 assay is a colorimetric test based on cleavage of the tetrazolium salt WST-1 into orange formazan by mitochondrial dehydrogenases in viable cells. The level of orange formazan produced increases when mitochondrial activity increases and can be quantified using an ELISA Reader (MWG-Biotech, Ebersberg, Germany) at 450 nm, with a reference wavelength at 690 nm.

### Quantitative real-time polymerase chain reaction assay

RNA was isolated from cultured cells inside the xCELLigence E-96 well plates after final impedance measurement and conventional 24-well plates using TRIzol reagent (Invitrogen, Carlsbad CA) as previously described [19]. RNA quantity and purity (A_260/280_) were measured using uQuant software. RNA was reverse transcribed into cDNA using 1 μg of mRNA and a High Capacity Reverse Transcription kit. (Invitrogen, Carlsbad, CA). Real-time polymerase chain reaction was performed using 10–100 ng of cDNA as a template and the StepOne Real-Time PCR System (Applied Biosystems, Foster City, CA). The resultant cycle threshold (Ct) values were normalized and analyzed using the standard curve method. TaqMan Gene Expression Assays were obtained for the following genes: type I collagen, type III collagen, tenascin-C, decorin, and scleraxis, relative to GAPDH as the endogenous control (Table 1). It was difficult to extract RNA from tenocytes exposed to 100% drug doses because they hardly proliferated in either culture system.

**Table 1:** Primers for reverse-transcription polymerase chain reaction to determine tenocyte gene expression

Gene	Primer sequence	Length (bp)
GAPDH	Sense: GAGTCCACTGGCGTCTCCAC Antisense: GGTGCTAAGCAGTTGGTGGT	188
Type I collagen	Sense: GGCCCAGAAGAACTGGTACA Antisense: GGCTGTTCTTGCAGTGGTAG	200
Type III collagen	Sense: CCAGGAGCTAACGGTCTCAG Antisense: CAGGGTTTCCATCTCTTCCA	103
Decorin	Sense: TGCTGTTGACAATGGCTCTC Antisense: GCCTTTTTGGTGTTGTGTCC	192
Tenascin-C	Sense: TCAAGGCTGCTACGCCTTAT Antisense: GTTCTGGGCTGCCTCTACTG	230
Scleraxis	Sense: CCTGAACATCTGGGAAATTTTAC Antisense: CGCCAAGGCACCTCCTT	111

### Statistical analysis

Each experiment was performed in triplicate. To compare cell indexes and readouts of WST-1 cell proliferation assays among different culture conditions, analysis of variance (ANOVA) followed by Tukey multiple comparison tests was used. Correlations between cell index results from the xCELLigence system and cell proliferation results from WST-1 assays in conventional culture were assessed with Pearson correlation analysis. Correlations between tenocyte gene expression upon culture in the xCELLigence system and conventional culture wells were assessed by Pearson correlation analysis, and Pearson’s correlation coefficients (*r*) were calculated. Differences were considered statistically significant when *p* values were less than 0.05. All statistical analyses were performed with SPSS 21.0 for Windows (SPSS Inc.).

## Results

### Determination of optimal cell density

**Fig. 3:**
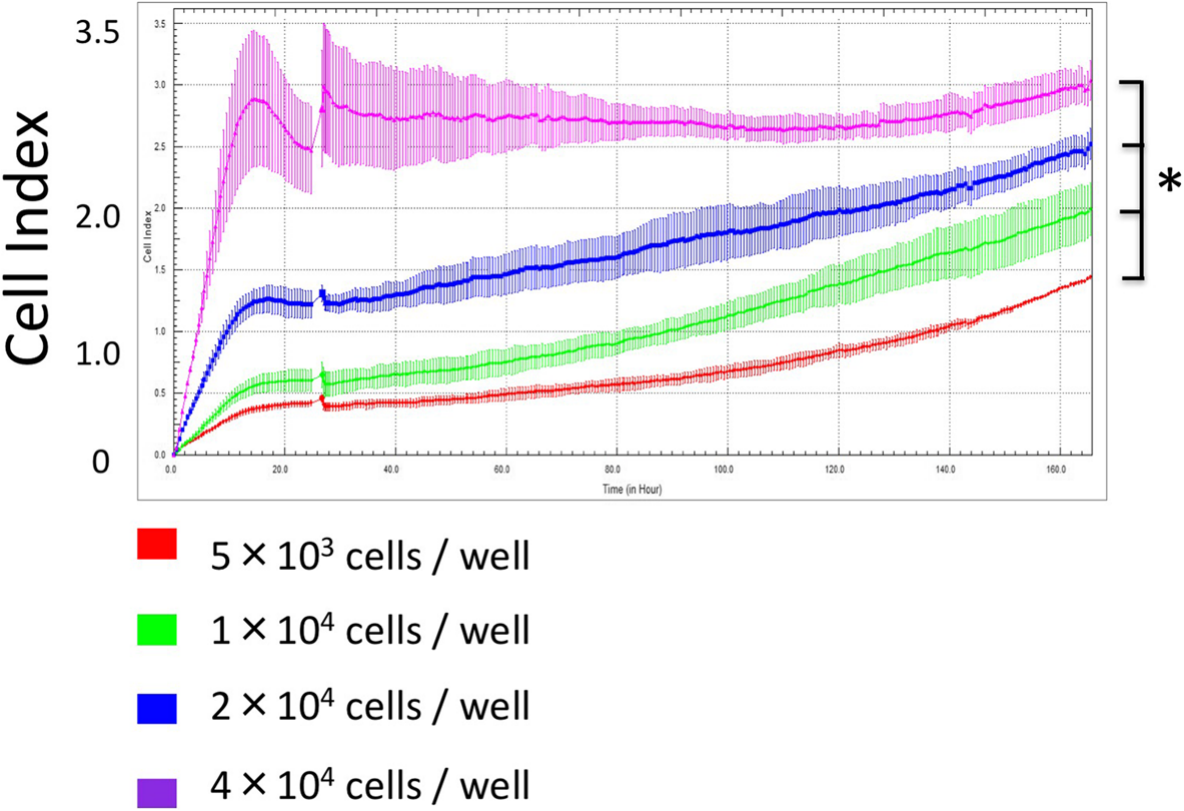
Four different tenocyte seeding densities. Cell index adhesion curves were obtained from different seeding conditions in real time with significant differences. *p < .05

Four different cell concentrations (5 × 10^3^ cells/cm^2^, 1 × 10^4^ cells/cm^2^, 2 × 10^4^ cells/cm^2^, and 4 × 10^4^ cells/cm^2^) were used in a pilot study to determine the optimal amount of cells to seed. For human tenocytes isolated from a torn supraspinatus, initial adhesion was rapid, indicated by a sharp increase in cell index over the first few hours after seeding. This was followed by a period of proliferation, indicated by a progressive increase in cell index. The proliferative phase was observed at all seeding densities and, as expected, occurred more slowly at lower seeding densities (Fig. 3). When 5 × 10^3^ tenocytes/cm^2^ were seeded, the cell index was 0.4 ± 0.01 after 24 h of cell adherence and increased to 1.4 ± 0.2 at the final time point (166 h). Cell indices from cell seeding densities of 1 × 10^4^ cells/cm^2^, 2 × 10^4^ cells/cm^2^, and 4 × 10^4^ cells/cm^2^ changed from 0.6 ± 0.01 to 2 ± 0.2, 1.2 ± 0.02 to 2.5 ± 0.12, and 2.45 ± 0.1 to 3 ± 0.16 at the final time point, respectively. Cell proliferation was detected in real-time according to increasing cell index. This indicated that human tenocytes could proliferate in the xCELLigence system and their real-time adhesion changes caused detectable and measurable impedance changes.

### Real-time cell index of tenocytes exposed to drugs

After the pilot study, a seeding density of 2 × 10^4^ cells/cm^2^ was used in the xCELLigence system. After 24 h of cell adherence, different concentrations of drugs were added to determine their real-time influence on cells. Tenocyte proliferation was determined by cell index in the xCELLigence system and WST-1 proliferation assays in conventional culture.

### Ketorolac tromethamine

**Fig. 4:**
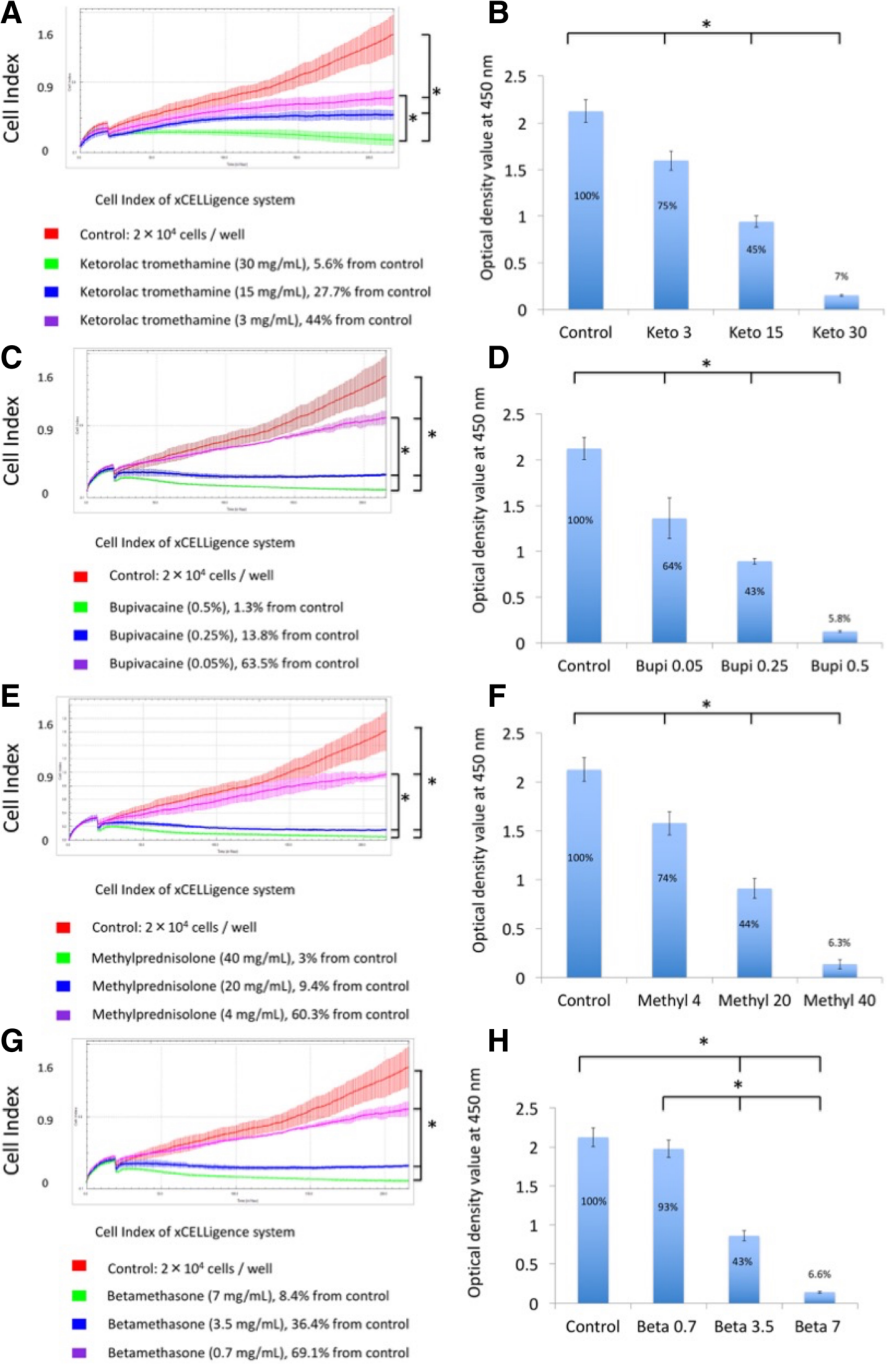
Cell index obtained from the xCELLigence system (**a**) and results of WST-1 assay upon tenocyte treatment with ketorolac tromethamine (**b**), bupivacaine (**c, d**), methylprednisolone (**e, f**), and betamethasone (**g, h**). *p < .05. Keto 3, 15, 30: ketorolac tromethamine 3 mg/mL, 15 mg/mL, 30 mg/mL; Bupi 0.05, 0.25, 0.5: bupivacaine 0.05, 0.25, 0.5%; Methyl 4, 20, 40: methylprednisolone 4, 20, 40 mg/mL; Beta 0.7, 3.5, 7: betamethasone 0.7, 3.5, 7 mg/mL

Cell index was measured upon exposure to 10 μL of 30, 15, or 3 mg/mL (100, 50, and 10% of clinical dosage) ketorolac tromethamine (Fig. 4a). The cell index of the untreated control group was 1.59 ± 0.3, which was decreased to 0.16 ± 0.01 upon exposure to 30 mg/mL ketorolac tromethamine (day 0, cell index right after exposure of drugs), the commonly used therapeutic dose. Cell index changed to 0.09 ± 0.01 at day 7 (final time point). When tenocytes were exposed to 50% diluted ketorolac tromethamine (15 mg/mL), Cell index was 0.14 ± 0.02 at day 0 and 0.44 ± 0.07 at day 7, which revealed the decreased cytotoxicity of diluted ketorolac tromethamine. When cells were exposed to the 10% dilution (3 mg/mL), the cell indices were 0.18 ± 0.02 and 0.7 ± 0.12 at day 0 and day 7, respectively. There were significant differences between the control group and the three concentrations of ketorolac tromethamine (*p* < .05). This suggested concentration-dependent cytotoxicity of ketorolac tromethamine. There were significant differences between groups except when 3 and 15 mg/mL (*p* = .30) and 15 and 30 mg/mL (*p* = .10) were compared. With xCELLigence system, detection of real-time cell proliferation was possible.

When the same dosages of ketorolac tromethamine were added in conventional 24-well plates, WST-1 assay results at day 7 revealed a concentration-dependent effect of ketorolac tromethamine on human tenocyte proliferation. The optical density values of control, 3, 15, and 30 mg/mL groups averaged 2.16 ± 0.12, 1.60 ± 0.1, 0.94 ± 0.06, and 0.15 ± 0.01 (Fig. 4b), respectively. Significant differences were found between groups (*p* = 0).

### Bupivacaine

Real-time changes in cell index were observed when tenocytes were exposed to 0.5, 0.25, and 0.05% (100, 50, and 10% of clinical dosage) bupivacaine (Fig. 4c). Cell index dropped to 0.14 ± 0.01 after exposure to 0.5% bupivacaine (day 0) and decreased to 0.02 ± 0.01 at day 7. This confirmed the cytotoxicity of 0.5% bupivacaine. When cells were exposed to 50 and 10% dilutions, the average cell indices changed from 0.18 ± 0.01 to 0.22 ± 0.02 (0.25%) and 0.28 ± 0.02 to 1 ± 0.07 (0.05%) at day 0 and day 7, respectively. The control group and the three different concentrations of bupivacaine showed significant differences in cell index (*p* < .05). Significance was not found between tenocytes exposed to 0.5 and 0.25% bupivacaine (*p* = .37), indicating that bupivacaine was cytotoxic even when diluted to 50% of the standard dosage.

When the same dosages were used in conventional system, WST-1 assays at day 7 revealed a concentration-dependent effect of bupivacaine on human tenocyte proliferation. The optical density of the control group, 0.05, 0.25, and 0.5% bupivacaine averaged 2.16 ± 0.12, 1.36 ± 0.22, 0.89 ± 0.03, and 0.13 ± 0.11 (Fig. 4d). Significant differences were found between groups (*p* < .05).

### Methylprednisolone

Real-time changes in cell index were observed upon cellular exposure to 40, 20, and 4 mg/mL (100, 50, and 10% of clinical dosage) methylprednisolone (Fig. 4e). The cell indices of tenocytes at day 0 and day 7 were 0.15 ± 0.01 and 0.05 ± 0.01 upon exposure to 40 mg/mL methylprednisolone. The average cell indices at day 0 and day 7 changed from 0.18 ± 0.01 to 0.15 ± 0.01 (20 mg/mL) and from 0.19 ± 0.02 to 0.96 ± 0.04 (4 mg/mL). There were significant differences between the control group and the three drug-treated groups (*p* < .05) except between 20 and 40 mg/mL group (*p* = .83). The results confirmed the cytotoxicity of methylprednisolone even upon a 50% dilution.

When the same dosages of methylprednisolone were added in conventional 24-well culture, WST-1 assays at day 7 revealed a concentration-dependent effect of methylprednisolone on human tenocyte proliferation. The optical density of the control group, 4, 20, and 40 mg/mL methylprednisolone averaged 2.16 ± 0.12, 1.58 ± 0.11, 0.91 ± 0.1, and 0.14 ± 0.05 (Fig. 4f), respectively. Significant differences were found between groups when tenocytes were exposed to different dosages of methylprednisolone (*p* < .05).

### Betamethasone

Real-time changes in cell index were observed upon cellular exposure to 7, 3.5, and 0.7 mg/mL (100, 50, and 10% of clinical dosage) betamethasone (Fig. 4g). Cell indices at day 0 and day 7 were 0.25 ± 0.02 and 0.13 ± 0.02 upon exposure to 7 mg/mL betamethasone. Average cell indices at day 0 and day 7 changed from 0.23 ± 0.03 to 0.57 ± 0.06 (3.5 mg/mL) and from 0.24 ± 0.03 to 1.1 ± 0.07 (0.7 mg/mL). There were significant differences between the control group and the three drug-treated groups (*p* < .05). Significant differences were also noted between the three concentrations of betamethasone treatment. This confirmed the concentration-dependent cytotoxicity of betamethasone.

When the same dosages of betamethasone were added in conventional 24-well culture, WST-1 assays at day 7 revealed the concentration-dependent effect of betamethasone on human tenocyte proliferation. The optical density of the control group, 0.7, 3.5, and 7 mg/mL betamethasone averaged 2.16 ± 0.12, 1.98 ± 0.11, 0.86 ± 0.07, and 0.14 ± 0.01 (Fig. 4h), respectively. Significant differences were found between groups except between the control and 0.7 mg/mL betamethasone group (*p* = .25).

### Comparison of different drugs

**Fig. 5:**
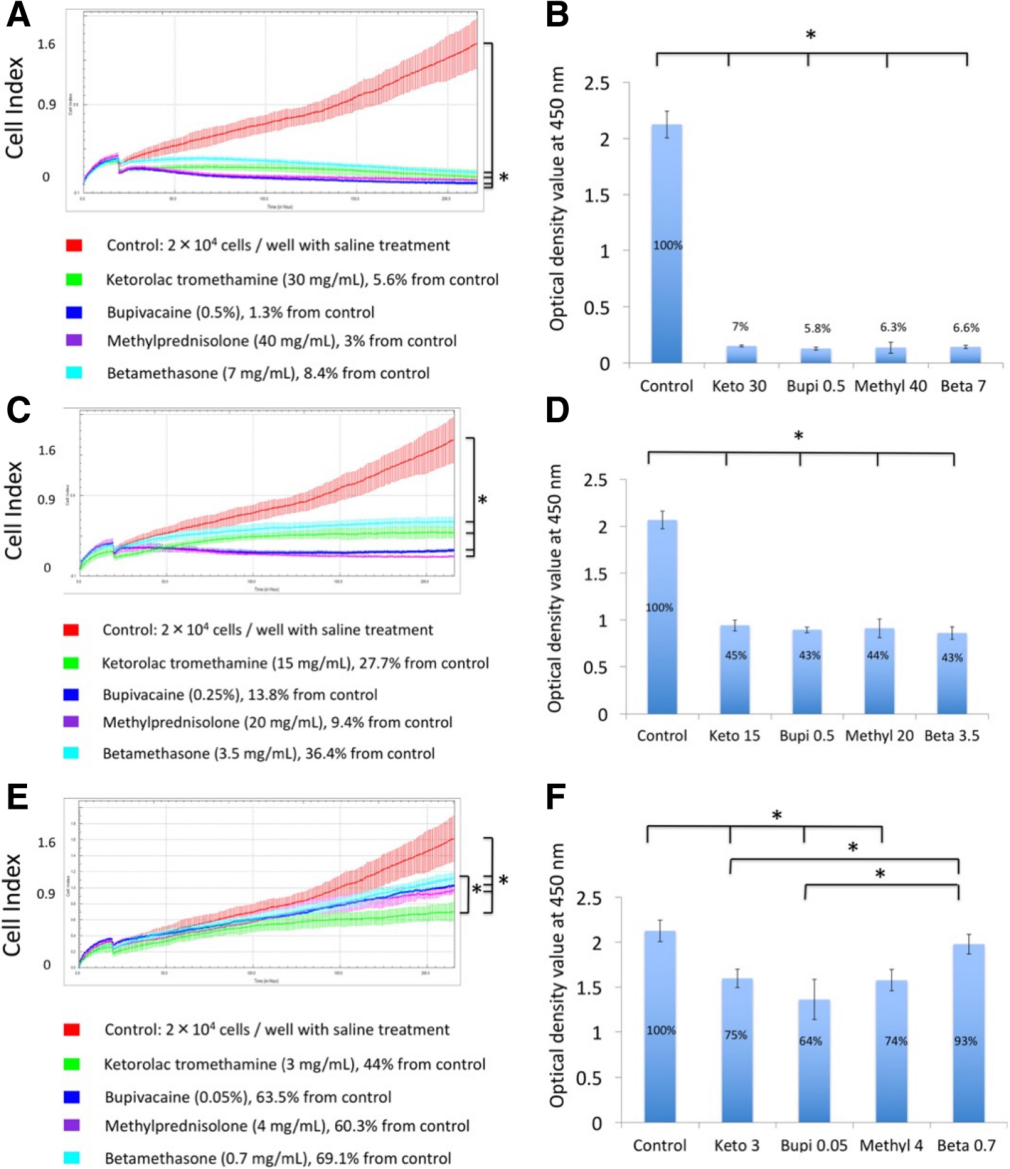
Comparison of tenocyte proliferation in the xCELLigence system and conventional culture system upon exposure to ketorolac tromethamine, bupivacaine, methylprednisolone, and betamethasone at 100, 50, and 10% of their respective clinical doses. Tenocytes exposed to 100% of ketorolac tromethamine, bupivacaine, methylprednisolone, and betamethasone in the xCELLigence system (**a**) and conventional cell culture (**b**). Tenocytes exposed to 50% of ketorolac tromethamine, bupivacaine, methylprednisolone, and betamethasone in the xCELLigence system (**c**) and conventional cell culture (**d**). Tenocytes exposed to 10% of ketorolac tromethamine, bupivacaine, methylprednisolone, and betamethasone in the xCELLigence system (**e**) and conventional cell culture (**f**). * *p* < .05

Cell index decreased immediately after drug exposure. The cytotoxicity of these drugs varied but all showed concentration-dependent effects. All four drugs at their 100% concentration exhibited significant cytotoxicity in both xCELLigence and conventional systems (Fig. 5a, b). There was no significance between the cytotoxicity of the four drugs, implying all of them had comparable cytotoxicity at their 100% concentration. Upon dilution to their 50% concentration, cytotoxicity did not decrease, except for betamethasone (*p* = .02) in xCELLigence, which implied a relatively decreased cytotoxicity in 50% diluted betamethasone compared to 100% betamethasone (Fig. 5c). However, this result was not observed in WST-1 (Fig. 5d). When comparing 10% drug concentrations in the xCELLigence system, significance was found between the control group and all four drugs, meaning that even at 10% of their therapeutic dose, cell viability decreased. Betamethasone at 10% dilution showed the least cytotoxicity. There was a significant difference between 10% betamethasone and ketorolac tromethamine treatment. WST-1 assay results indicated significant differences between the control group and 10% ketorolac tromethamine, bupivacaine, methylprednisolone, but not betamethasone, confirming the decreased cytotoxicity of 10% betamethasone observed in the xCELLigence system. Among ketorolac tromethamine, bupivacaine, and methylprednisolone diluted to 10% of their therapeutic dose, there were no significant differences in cytotoxicity (Fig. 5f), implying that 10% diluted betamethasone had the least cytotoxicity of the drugs tested.

### Effect of different drugs on matrix gene expression

**Fig. 6:**
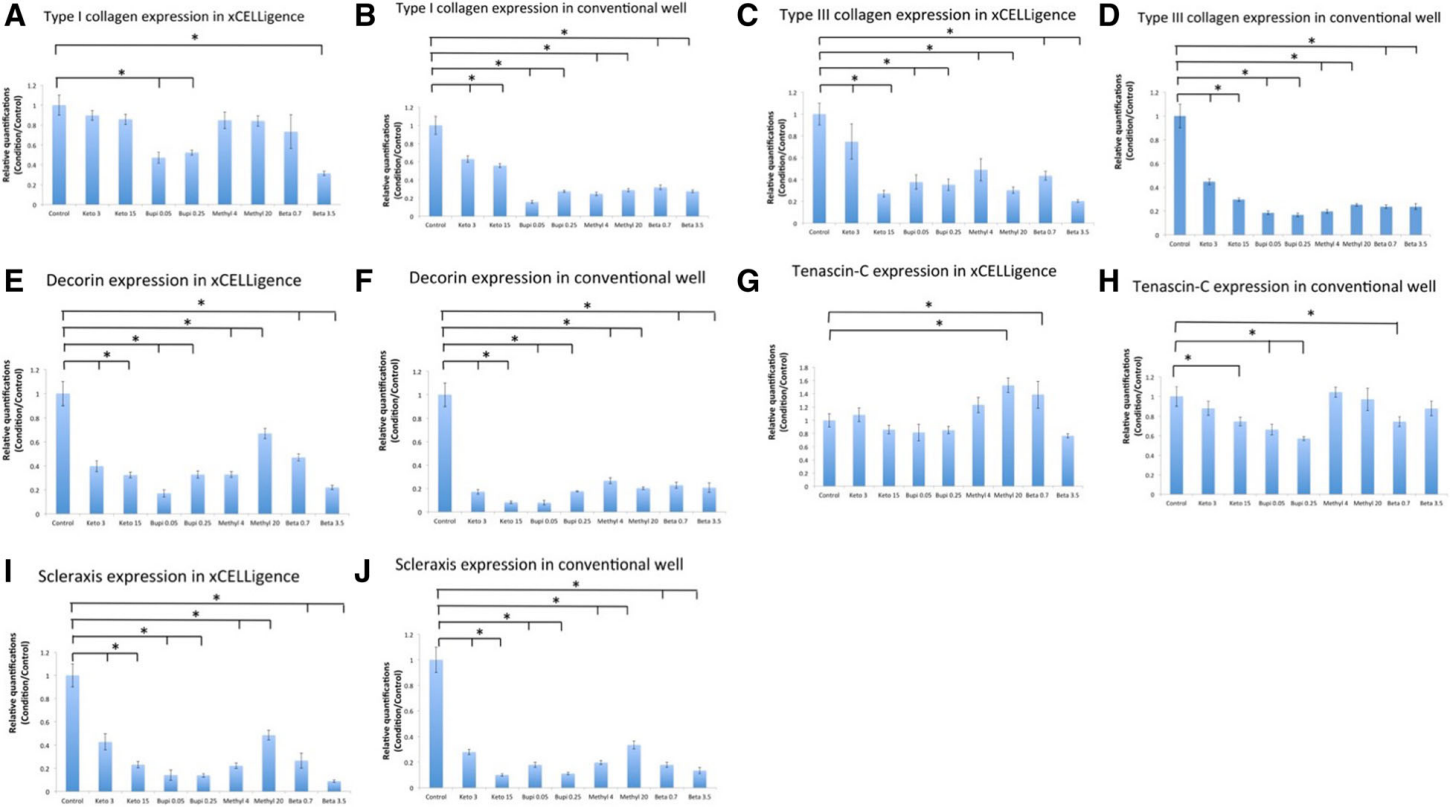
Tenocyte gene expression upon culture in the xCELLigence system and conventional culture system. Type I collagen expression in the xCELLigence system (**a**) and conventional culture system (**b**). Type III collagen expression in the xCELLigence system (**c**) and conventional culture system (**d**). Decorin expression in the xCELLigence system (**e**) and conventional culture system (**f**). Tenascin expression in the xCELLigence system (**g**) and conventional culture system (**h**). Scleraxis expression in the xCELLigence system (**i**) and conventional culture system (**j**)

For tenocytes cultured in the xCELLigence system, 0.25 and 0.05% bupivacaine and 3.5 mg/mL betamethasone treatment significantly decreased type I collagen gene expression compared with the control at day 7 (Fig. 6a). All concentrations of all drugs decreased type I collagen gene expression at day 7 compared to control in conventional culture (Fig. 6b). For tenocytes in xCELLigence and conventional culture, all drugs decreased the gene expression of type III collagen, decorin, and scleraxis compared to expression in control cells at day 7 (Fig. 6c–j). For tenocytes cultured in the xCELLigence system, 20 mg/mL methylprednisolone and 0.7 mg/mL betamethasone significantly increased tenascin gene expression at day 7 (Fig. 6g). However, 15 mg/mL ketorolac tromethamine, 0.05% bupivacaine, 0.25% bupivacaine, and 0.7 mg/mL betamethasone significantly decreased tenascin gene expression at day 7 in conventional culture (Fig. 6).

### Correlation between cell index from the xCELLigence system and WST-1 proliferation assays

**Fig. 7:**
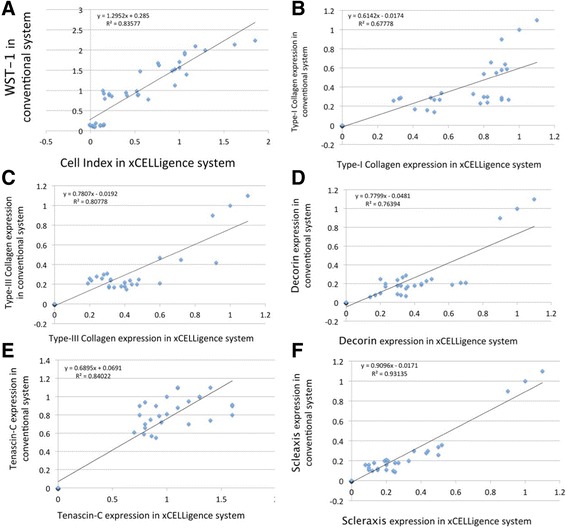
Correlation of results from the xCELLigence system and the conventional culture system. **a** Correlation of cell index in xCELLigence and WST-1 assay results. Correlation of type I collagen expression (**b**), type III collagen expression (**c**), decorin expression (**d**), tenascin-C expression (**e**), and scleraxis expression (**f**) upon tenocyte culture in the xCELLigence system or conventional culture

Each experiment was performed in triplicate. A high, positive correlation between cell index from the xCELLigence system and WST-1 proliferation assay results was observed (Pearson’s correlation [*r*] = 0.914, *p* = 0) (Fig. 7a, Table 2).

**Table 2:** Correlations between tenocyte gene expression upon culture in the xCELLigence system or conventional culture wells

	Person correlation coefficient	*p* value
Cell proliferation: cell index of xCELLigence and result of WST-1	0.914	0
Gene expression of type I collagen in xCELLigence and conventional culture	0.823	0
Gene expression of type III collagen in xCELLigence and conventional culture	0.899	0
Gene expression of decorin in xCELLigence and Conventional culture	0.917	0
Gene expression of tenascin-C in xCELLigence and conventional culture	0.874	0
Gene expression of scleraxis in xCELLigence and conventional culture	0.965	0

### Correlation between tenocyte gene expression after culture in the xCELLigence system or conventional culture wells

A positive correlation in gene expression was observed between tenocytes cultured in the xCELLigence system and conventional culture wells. Pearson’s correlation [*r*] of type I collagen, type III collagen, tenascin-C, decorin, and scleraxis were as follows: type I collagen: [*r*] = 0.823, *p* = 0; type III collagen: [*r*] = 0.899, *p* = 0; tenascin-C: [*r*] = 0.917, *p* = 0; decorin: [*r*] = 0.874, *p* = 0; and scleraxis: [*r*] = 0.965, *p* = 0. (Fig. 7b–f, Table 2).

Correlations between tenocyte gene expression upon culture in the xCELLigence system and conventional culture wells.

## Discussion

Tendons are composed of tenocytes in a three dimensional extracellular matrix network, and these cells synthesize major components of tendons that give them biomechanical properties and maintain their structure [20, 21]. However, tenocytes are highly differentiated cells with limited potential to replicate and proliferate, the instinct healing ability of tendon is poor [22, 23]. Therefore, negative effects on tenocyte viability should be minimized when treating tendon injuries [24].

Local injections of NSAIDs, anesthetics, and corticosteroids are commonly used to treat tendinopathies [1]. This study found that ketorolac tromethamine, bupivacaine, methylprednisolone, and betamethasone had a dose-dependent detrimental effect on tenocytes proliferation. There is considerable debate over the risk-to-benefit ratio of using these drugs for reducing tendon pain while potentially compromising tissue strength and repair capabilities. Sung et al. exposed cultured human rotator cuff tenofibroblasts to two different concentrations of three local anesthetics at various time points [3]. They found different types and doses of anesthetics resulted in different cytotoxicity, which was dependent on exposure time and concentration. However, studies regarding the dose-dependency of adverse effects of these drugs on individual tendon are lacking, let alone the real-time changes in tenocyte proliferation upon exposure to them.

Traditional cell studies observed cell proliferation in conventional culture wells. While these methods are well-accepted, they require large numbers of cells, large volumes of reagents, and are limited in their accessibility for high resolution and time-lapse imaging [25]. Culture media should be changed periodically, which is labor consuming and leads to high experiment cost. Hung et al. developed a microfluidic cell culture array fabricated by soft-lithography technology and replicate molding. This system was designed to maintain and monitor cells continuously while providing a stable microenvironment. In this sterile cell culture microenvironment, preparation of multiple assay conditions and long-term continuous monitoring methods in an integrated device was successful [6]. Moreover, an electronic circuit was integrated into the microfluidic chip for electrical stimulation and recording of cellular responses. [9] Microfluidic cell culture systems have been employed commercially to create more biologically relevant cellular microenvironments and higher throughput analysis platforms of cell behavior compared to conventional techniques [8]. Cell-based biosensors and drug screening technology have been used to monitor cell behavior in the liver [26], human hepatocytes [27], lung [28], heart [29], blood brain barrier [30], gut [31], and kidney [32]. For tenocytes, Dolkart et al. [33] utilized microfluidic systems to analyze rat tenocyte adhesion with an impedance-based instrument system (iCELLigence, Roche). They seeded 1 × 10^4^ cells/cm^2^ into 8-well plates.

In a pilot study, we found the appropriate seeding density for human tenocytes into the xCELLigence E-96-well plate was 2 × 10^4^ cells/cm^2^. When 4 × 10^4^ tenocytes/cm^2^ were used, the proliferation slope was not as steep as the slope observed upon seeding 2 × 10^4^ cells/cm^2^. Another concern with conventional methods stemmed from the fact that passaging tenocytes multiple times causes phenotypic drift. This posed a problem in tenocyte research because freshly cultured tenocytes are not typically available in sufficient amounts. Studies have shown the potential for tenocyte phenotypic drift after prolonged maintenance in monolayer cell culture [34–36]. Changes are observed in growth characteristics and alterations to the composition of the extracellular matrix. Mazzocca et al. reported that types I and III collagen gene expression significantly decreased after two passages. Decorin and tenascin-C gene expression in tenocytes exhibited a decreasing trend with increased passages. Types I and III collagen and decorin protein levels decreased after four passages [19]. Therefore, only cells within the first three passages should be used for in vitro monolayer cell models [19]. If 4 × 10^4^ cells/cm^2^ are used for experiments, more tenocytes should be prepared. Phenotypic drift would be inevitable because multiple passages would be needed to prepare a sufficient amount of tenocytes.

There was fine line between anti-inflammatory/analgesic effect and cytotoxicity of NSAIDs, anesthetics, and steroids. One concern was the exact timing of cytotoxicity resulting from treatment with these drugs. With the use of xCELLigence, we could see real-time changes in tenocyte proliferation immediately after drug treatment. This data could aid in determining the optimal timing of local injection and help to avoid overdose of these drugs. The results showed a strong, positive correlation between cell index and WST-1 assay results. Tenocytes cultured in both systems showed similar expression patterns of types I and III collagen, tenascin-C, decorin, and scleraxis. Utilizing the xCELLigence system instead of conventional culture methods can allow clinicians to determine the optimal dose of drugs before injection.

There are still limitations and weaknesses in this study. First, this in vitro model may not represent in vivo conditions. However, conventional cell culture methods cannot represent true in vivo conditions either. Microfluidic technology is designed to provide defined spatiotemporal conditions to cells with user-controlled input, minimizing differences between in vitro models and complex in vivo microenvironments. Since the result revealed that both systems shared similar results, this technology may replace conventional cell culture. Second, tenocytes used in this study were isolated from a 64-year-old female. Cells from elderly donors are inferior, exhibiting a slower cell metabolism [37]. Tenocytes isolated from patients of different ages may present different characteristics. Therefore, the same dose and concentration of drugs for local injections should not be used for different people. In Sung’s study,^3^ 6 local anesthetic subgroups were employed with two concentrations and 11 exposure times. A large number of tenofibroblasts was needed for such experiment but only a small amount of diseased tissue could be retrieved during surgery. If a large number of tenocytes is needed to complete a study, cells should be passaged multiple times, causing phenotypic drift. This phenomenon will skew in vitro study results. With the help of the xCELLigence system with high throughput data, the least toxic dose of drugs can be determined before injection into each individual with fewer cells compared to conventional methods if the exposed cells are designed to be retrieved to have further biological study like PCR. Third, only one patient with a torn supraspinatus tendon was enrolled in present study. Cells from individuals with different tendinopathy locations and stages may yield different results. The objective of this study was to determine if the xCELLigence system could serve as a relatively easy-to-handle platform to replace conventional cell culture methods. The same protocol may be applied for further animal or human studies.

The advantage of this study is that microfluidic technology provides an opportunity to apply optimized dose of drugs in treating tendinopathies because of its relatively simpler method for cell culture and capability of monitoring drugs effects on cell proliferation in real-time fashion. With the help of the xCELLigence system, a fine balance between the analgesic and cytotoxic effect of injected drugs can be achieved. On the other hand, this system may provide a platform to determine the ideal timing for introducing growth factors that are believed to help tenocyte proliferation. The objective of this study was to share our experience and highlight applications of the xCELLigence system, especially for researchers currently using conventional end-point assays to evaluate tenocyte proliferation. This system represents a powerful platform for screening drug toxicity.

## Conclusions

Human tenocytes isolated from a torn supraspinatus can proliferate successfully inside xCELLigence system at an optimal seeding density of 2 × 10^4^ cells/cm^2^. This made real-time monitoring tenocyte proliferation possible. Cellular responses varied when tenocytes were exposed to different concentrations of ketorolac tromethamine, bupivacaine, methylprednisolone, and betamethasone. Tenocyte proliferation and gene expression was comparable between the xCELLigence system and the conventional culture system. The data could be further applied to determine the optimal dosages and timing of ketorolac tromethamine, bupivacaine, methylprednisolone, and betamethasone when local injections of are warranted.

## Abbreviations

CI: Cell index
ELISA: Enzyme-Linked ImmunoSorbent Assay
NSAIDs: Non-steroid anti-inflammatory drugs
PCR: Polymerase chain reaction
